# Native proteins from *Galdieria sulphuraria* to replace fetal bovine serum in mammalian cell culture

**DOI:** 10.1007/s00253-025-13507-0

**Published:** 2025-05-10

**Authors:** Hanna Eisenberg, Svenja Hütker, Felicitas Berger, Imke Lang

**Affiliations:** Institute EcoMaterials, Bremerhaven University of Applied Sciences, An Der Karlstadt 8, 27568 Bremerhaven, Germany

**Keywords:** *Galdieria sulphuraria*, Native protein, Enzyme activity assay, Mammalian cell culture, Fetal bovine serum

## Abstract

**Abstract:**

The use of fetal bovine serum (FBS) in cell culture applications causes high costs and unacceptable animal suffering when FBS is extracted from fetal calves. Despite efforts, the exact composition of FBS still remains partially unresolved. Native proteins in FBS, such as growth factors, and their binding to cell receptors seem to be crucial for cell proliferation and differentiation. Recently, algal extracts with high protein content were considered to reduce the FBS demand. Algae extracts yielded promising results as growth serum in mammalian cell culture. Nevertheless, the dependence on residual FBS and the undefined composition of algae extracts are challenges. In this study, we aimed to yield highly concentrated extracts of native proteins from mixotrophically grown *Galdieria sulphuraria* to replace FBS in mammalian cell culture. Crude extracts and native proteins were concentrated by ammonium sulfate precipitation, and all extracts underwent heat inactivation (HI) for selective protein inactivation. The remaining proteins’ native conformation was verified by enzyme activity assays. All extracts were used to replace FBS during the cultivation of Chinese hamster ovary (CHO) cells, and proliferation was tested. We found that *G. sulphuraria* crude and protein extracts depended on HI to promote CHO cell growth to a similar extent as FBS. CHO cells grown with 5% or 10% heat-treated algal extracts had a relative proliferation of 260 to 230% compared to FBS controls with 210% and 300%, respectively. We anticipate our findings will help replace FBS in mammalian cell culture, increasing sustainability and consumer acceptance.

**Key points:**

- *Reproducible production of FBS substitutes from microalgae is a key to sustainable mammalian cell culture*.
- *Heat-treated native protein extracts of G. sulphuraria lead the way to new media additives.*
- *Identification of effective molecules is mandatory for the composition of a new culture medium.*

**Supplementary Information:**

The online version contains supplementary material available at 10.1007/s00253-025-13507-0.

## Introduction

Cell and tissue culture is becoming increasingly important for basic research, as well as biotechnological and medical applications. Mammalian cell and tissue cultures are crucial for the evaluation of toxic substances and can reduce and replace the necessity of animal testing (Zhu et al. 2024). Chinese hamster ovary (CHO) cells represent the most common expression platform for the large-scale production of therapeutic proteins, such as monoclonal antibodies (Dhara et al. 2018). Recently, cell culture has also established itself as an effective solution for culture-based fish and meat products entering the market (Eibl et al. 2021; Quek et al. 2024).

A limitation for in vitro cultivation of animal cells is their need for growth factors. The most common supplement is fetal bovine serum (FBS), which is rich in native proteins. For example, growth factors and hormones bind to cell receptors according to the key-lock principle and help the cell to remain within the cell cycle or prevent apoptosis (Wang 2021). FBS is usually added as a sterile filtered extract so that proteins and other components do not denature and remain in their natural state (Francis 2010).

There are substantial ethical and scientific concerns about the use of FBS in cell culture. The product is generated under precarious conditions, and the quality of the serum fluctuates between batches (van der Valk et al. 2004). Furthermore, there are safety concerns due to the risk of contamination with viruses or *Mycoplasma* species, respectively (Ozturk and Hu 2005). Finally, the price of FBS has doubled within the last 5 years, ranging from 1000 to 2000$ L^−1^ depending on the source and quality (ThermoFisher Waltham, MA, USA, and Gibco, Grand Island, NY, USA). Thus, a chemically defined serum-free medium would allow for a stable and reproducibly high productivity for the large-scale production of biopharmaceuticals or other cell-based therapeutics. In the search for alternatives, different chemically synthesized and bio-derived products were identified as possible replacements for FBS, but these are not standard serum-free media, and each cell type requires its own composition of the medium (van der Valk et al. 2018; Subbiahanadar Chelladurai et al. 2021; Quek et al. 2024).

The composition of commercially available serum-free media is often not disclosed, prompting scientists to seek accessible alternatives (Gstraunthaler and Lindl 2017). Human thrombocyte lysates, heat-inactivated coelomic fluid from earthworms, and sericin from raw silk have contributed to replacing FBS (Terada et al. 2002; Cao and Zhang 2015). So far, plant-based extracts have not matched FBS’s effectiveness in promoting cell growth (Pazos et al. 2004). However, research into the use of microalgae extracts in cell culture media appears to be worthwhile. Microalgae can grow in seawater and on wastewater nutrients, with higher productivity per unit area (Zakaria and Kamal 2016). They are used for biofuels, cosmetics, and food supplements (Occhipinti et al. 2024). In the latter, their ability to produce large quantities of digestible protein may render them a major source of protein to feed a growing population. The use of algal extracts in cell culture has been conceived for more than a decade (Song et al. 2012; Okamoto et al. 2020; Amorim et al. 2021; Defendi-Cho and Gould 2023; Dong et al. 2023; 2024; Sibinčić et al. 2024). Hot water extracts of *Chlorella vulgaris* (CVE) increased IGF-1 receptor expression and phosphorylation of focal adhesion kinase and Src in rat intestinal epithelial cells (Song et al. 2012). CVE also increased MAPK and PI3 K/Akt pathway when tested with 10% FBS. Later, FBS reduction to 2% in cell culture medium was achieved using *C. vulgaris* hot water extracts, referred to as *Chlorella* growth factor (CGF) (Ng et al. 2020). The major CGF components were proteins and carbohydrates, with the protein fraction being highest at 67%. A hot water extract from *Auxenochlorella pyrenoidosa* (APE) revealed to be effective in fish cell cultures and promoted cell proliferation in low-serum culture medium; a complete FBS replacement was not possible (Dong et al. 2023; 2024). In a similar approach, *Chlorococcum littorale* compounds soluble in water (CW) showed to be an effective FBS replacement in three different mammalian cell lines (Ghosh et al. 2024). Additionally, extracts obtained from the cyanobacterium *Anabaena* PCC 7120 were used as a media supplement to cultivate mouse and quail muscle cells (Ghosh et al. 2023). In a screening study, water extracts of *Arthrospira platensis* and *Dunaliella tertiolecta* successfully replaced up to 90% of FBS in cell culture medium (Sibincˇic et al. 2024). Published results indicate that the water-soluble fraction of microalgae promotes growth in mammalian and fish cell cultures. However, the composition of these algae-derived replacements is largely unidentified. To develop a defined serum substitute, it is crucial to identify the active algae fractions and analyze individual molecules and their targets in mammalian cells.

In this study, we set out to produce standardized, highly concentrated native protein extracts from microalgae to reliably replace FBS in cultured mammalian cells. We selected *Galdieria sulphuraria*, a protein-rich, thermo-acidophilic red alga, for high biomass production. This well-characterized species can produce highly concentrated protein extracts and grow on various organic carbon sources in a (photo)bioreactor (Schmidt et al. 2005; Sloth et al. 2017; Abiusi et al. 2022; Lang et al. 2022). Optimal growth conditions for *G. sulphuraria* are a pH of 1–2.5 and temperatures between 35 and 45 °C (Gross and Schnarrenberger 1995). This reduces contamination risk and cooling costs in closed photobioreactors (PBRs). Additionally, *G. sulphuraria* is scalable for industrial production and is being evaluated as a novel food ingredient (Fermentalg, Libourne, France; Galdieria Blue Extract, 26.03.2025, 10:27 a.m.). Here, we set a mixotrophic cultivation of *G. sulphuraria* (strain 074G) to gain a high biomass yield. We recovered native protein fractions and validated the stability of native proteins by enzyme activity assessment of typical cytosolic and chloroplast enzymes. Then, raw algae extracts, as well as native and heat-inactivated protein fractions, were tested as possible substitutions for FBS in mammalian cell culture.

## Material and methods

### Strain and culture conditions

The strain *G. sulphuraria* 074G was kindly provided by Christine Oesterhelt (also available as CCCryo 120–00, Fraunhofer Institute for Cell Therapy and Immunology, Postdam-Golm, Germany). For all experiments, the culture medium was M Allen (Allen 1959) with pH 2.5 and supplemented with 0.28 M glucose and 37.84 mM additional ammonium sulfate for mixotrophic growth. Pre-cultures were grown in 50 mL medium in a 100-mL sterile Erlenmeyer flask. The cultures grew mixotrophically at an average photon flux density (PFD) of 77 µmol photons m^−2^ s^−1^ provided by fluorescent bulbs with a 12:12 h light/dark cycle on a shaker (200 rpm) in a 40 °C incubator. After 1 week, the pre-cultures were transferred into a 1-L bottle with 900 mL medium. Cultures grew at 358 µmol photons m^−2^ s^−1^ with a 12:12 h light/dark cycle and at 200 rpm (9-cm stir bar) at room temperature (RT). Once the cultures reached an optical density (OD) of around 6 at 750 nm, inoculation of a 6-L flat-panel airlift PBR (LS6 Subitec GmbH, Köngen, Germany) followed. The experiments were conducted under 24 h or 12 h illumination with a 320-W light-emitting diode (LED) at an average PFD of 150 µmol photon m^−2^ s^−1^ from one side. Mixing in the PBR was achieved by a continuous airflow of 150 NL h^−1^. The culture temperature was 38 °C. For inoculation, medium was pumped through a membrane filter into the reactor. Then, the pre-culture was pumped into the reactor to reach a starting culture density of approximately 0.5 g L^−1^. The experiments were conducted in batch mode and lasted for 5 days.

### Determination of cell growth

Growth of *Galdieria* cultures was monitored by measures of OD at 750 nm and biomass dry weight (DW) in g L^−1^. For DW, a respective volume of culture suspension was filtered through pre-dried glass-fiber round filters, Whatman™, 45 mm diameter (Cytiva, Global Life Sciences Solutions USA LLC, Marlborough, MA, USA) and then washed twice with distilled water. The filter was placed in an aluminum tin and dried at 50 °C for 3 days before the weight was taken. The values of OD and DW were used for a calibration between the two measures. The conversion factor was 1 OD mL^−1^ = 0.468 g dry biomass L^−1^ for *G. sulphuraria* 074G under the given mixotrophic conditions.

### Optimization of cell disruption

The biomass was harvested by centrifugation at 4302 g for 15 min at 4 °C. The supernatant was discarded, and the cell pellet was washed twice with distilled water. The cell disruption was carried out by using a bead mill (Mini-BeadBeater 96, Biospec products, INC., Bartlesville, USA) for 30 s at 2100 rpm, with 2 min of cooling at − 20 °C in between. To achieve cell lysis and optimal recovery of the soluble protein, the properties of the wet biomass (frozen, thawed) and the size and quantity of the glass beads (GB) were investigated. For the first approach, 3 g of thawed biomass was resuspended in 30 mL of 50 mM phosphate buffer saline (PBS) + 500 mM NaCl pH 7.0 (PBS + NaCl). GB of 0.25–0.5 mm or 0.75–1 mm were added to the sample at a ratio of 1:10 (w/w). To the frozen wet biomass, an equal weight of GB was added, and only after cell disruption, the biomass was resuspended in 10 mL PBS + NaCl buffer. In the second approach, thawed biomass was used to study GB ratios of 1:10 (w/w) and 1:5 (w/w) as well as three consecutive runs of cell disruption with a GB ratio of 1:3.3 (w/w) in 10 mL buffer. After two cycles of bead milling, the Falcons for the consecutive preparation were centrifuged at 4302 g for 10 min at 4 °C, the supernatant was collected, and the cell pellet was resuspended again in 10 mL PBS + NaCl buffer. This was repeated two more times, for a total of three cycles.

### Protein extraction and quantification

The harvest of biomass was conducted as described for the cell disruption optimization experiments. After washing the pellet, 3 g of fresh biomass was resuspended in 30 mL of PBS + NaCl. With the addition of 10 g of GB (0.25–0.5 mm), cells were disrupted in six consecutive runs of 30-s bead milling (Mini-BeadBeater 96, Biospec products, INC., Bartlesville, USA) at 2100 rpm, with 2-min cooling in between. After every second run, three times in total, the biomass was centrifuged at 4302 × g for 10 min at 4 °C. Supernatant was collected and stored on ice, and the pellet was resuspended in 10 mL PBS + NaCl. After centrifugation of the collected supernatant at 11,000 × g for 2 h at 4 °C, aliquots of the supernatant (crude extract) were further ultra-centrifuged at 61,973 × g for 20 min at 4 °C (Sigma 3-30 KS, Sigma Laborzentrifuge GmbH, Osterode am Harz, Germany). Native proteins were precipitated by the addition of ammonium sulfate (100% saturated solution) up to a saturation of 60% at 4 °C and incubation at 4 °C overnight. The proteins were recovered by centrifugation (11,000 × g, 2 h, and 4 °C), resuspension in 50 mM PBS buffer pH 7, and subsequent desalination using a PD-10 desalting column/Sephadex G-25 resin desalting column (Cytiva, Global Life Sciences Solutions USA LLC, Marlborough, MA, USA). If the extracts were heat-inactivated, they were incubated at 70 °C for 20 min, and the supernatant was collected after centrifugation (13,000 × g, 20 min, and 4 °C). The protein content of the crude extract and protein eluants were assayed following Bradford and Lowry (Lowry et al. 1951; Bradford 1976). Total protein after culture harvest was followed by the protocol of de Marsac and Houmard (1988) with the subsequent Lowry assay protocol for protein quantification.

### SDS-PAGE

Sodium dodecyl sulfate–polyacrylamide gel electrophoresis (SDS-PAGE) was performed to obtain a rough insight into the protein content of the algae extracts (Laemmli 1970). All samples were mixed with 4:1 (v/v) 4 × SDS sample buffer (Roti-Load 1, Carl Roth GmbH + Co. KG, Karlsruhe, Germany) and incubated at 95 °C for 5 min. Insoluble protein was first resuspended in an equal volume of deionized water corresponding to the total volume of the respective crude extract. Either 20 µL of undiluted samples were loaded or a protein amount of 10, 30, or 50 µg, and protein marker (PageRuler Prestained Protein Ladder, Thermo Scientific, Darmstadt, Germany) to a 10% acrylamide gel. Protein separation was performed in a running buffer containing TRIS base (0.25 M), glycine (1.92 M), and SDS (1% (w/v)) (Rotiphorese 10 × SDS-PAGE, Carl Roth GmbH + Co. KG, Karlsruhe, Germany) for approx. 50 min at 25 mA per gel. The gel was subjected to the fixing solution (40% v/v methanol, 10% acetic acid) and stained (0.2% (w/v) with Serva blue G-250 in 90% methanol mixed 1:1 to acetic acid) and subsequently destained (methanol/acetic acid/H_2_O (2:1:7)).

### Enzyme activity assays

To determine the specific activities of enzymes, 10 or 20 µL of the extracts were combined with pyruvate kinase (PK) and phosphoenolpyruvate carboxylase (PEPC) reaction mixtures in wells of microtiter plates at room temperature. The reaction mixture for the PEPC activity contained a total volume of 300 µL 10 mM MgCl, 30 mM NaHCO_3_, 5 mM phosphoenolpyruvic acid (PEP), 0.5 U mL^−1^ magnesium hydroxide (MDH), 0.2 mM NADH, and 100 mM HEPES/NaOH buffer, pH 7.4. The increase of absorbance at 340 nm was monitored over 15 min. The reaction mixture for the PK activity contained a total volume of 300 µL 10 mM MgCl_2_, 50 mM KCl, 1 mM ADP, 5 mM PEP, 2 U mL^−1^ lactate dehydrogenase (LDH), 0.2 mM NADH, 50 mM Tris/HCl buffer, and pH 8.0. The activities obtained were normalized to the protein content per well to calculate the specific activities.

### Mammalian cell culture and 3-[4,5-dimethylthiazol-2-yl]−2,5 diphenyl tetrazolium bromide (MTT) assay

CHO cells (CHO-K1| 603480, Cell lines Service GmbH, Eppelheim, Germany) were cultivated in 75-cm^2^ flask until sub-confluency of approximately 80% in proliferation medium (Th. Geyer GmbH and Co. KG, Höxter, Germany, BioWest Ham’s F-12) containing 10% FBS (Sigma-Aldrich, Taufkirchen, Germany, Lot. BCCC4867, F0804) and 1% penicillin–streptomycin (Sigma-Aldrich, Taufkirchen, Germany). They were incubated at 37 °C with 5% CO_2_ and renewed every second day. Subcultivation was achieved by removing the medium and using 2.5% (w/v) trypsin (Sigma-Aldrich, Taufkirchen, Germany) in PBS (Carl Roth GmbH + Co. KG, Karlsruhe, Germany) at 37 °C. Trypsinization was stopped by the addition of 10 mL of proliferation medium and centrifugation at 800 g for 7 min. After resuspension in fresh proliferation medium, 10,000 viable cells were seeded per well of a 96-well plate for the proliferation assay. Ten percent FBS and 5% FBS were used as positive controls. For assessing changes in proliferation, 5 µL of Rotitest Vital (Carl Roth GmbH + Co. KG, Karlsruhe, Germany) was applied to each well using a multichannel pipette. Cells were then incubated for 90 min, and the absorption was measured at 450 nm using Gen5 Microplate Reader and Imager Software by BioTek and Epoch Microplate Reader (Biotek Instruments, Winooski, VT, USA). For the remaining plates, the medium was changed every 24 h during the experiment.

## Results

### Protein recovery from *G. sulphuraria* biomass

**Fig. 1:**
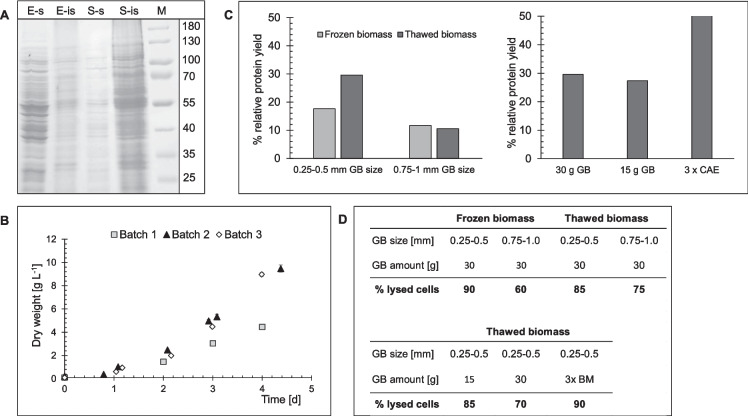
Optimization of cell disruption and protein extraction from wet biomass of *G. sulphuraria*. **A** SDS page showing soluble (s) and insoluble (is) protein fractions in the CE (crude extract) of biomass harvested in exponential (E) and stationary (S) phases. **B** Growth of three batches of *G. sulphuraria* in a 6 L flat-panel PBR under 12 h (batch 1) and 24 h (batches 2 and 3) illumination. Cells were grown in the presence of 0.28 M glucose and 37.84 mM ammonium sulfate. Growth is expressed as DW (g L^−1^) over the course of 5 days. **C** Cell disruption optimization via bead mill, varying GB size between 0.25 and 0.5 mm and 0.75 and 1 mm on either frozen biomass or thawed biomass. Protein yields relative to DW indicated cell disruption. The results shown represent one test run; a repetition showed comparable results. The second experiment represents the cell disruption using 0.25–0.5 mm GB in different amounts, 15 and 30 g, and three consecutive extraction cycles of bead milling (BM). **D** Table summarizing the microscopically observed percentage of lysed cells for the different cell disruption conditions

To establish biomass for protein extraction, *G. sulphuraria* 074G was cultured mixotrophically in batch mode in a PBR. Batches that were grown until the stationary phase yielded 74.3 g dry biomass (data not shown). This biomass showed a large insoluble protein fraction after cell disruption (Fig. 1A). Biomass harvested during the late exponential or linear phase had a larger fraction of soluble protein (Fig. 1A). Thus, subsequent cultivations were harvested at approximately 10 g L^−1^. Continuous illumination for batches 2 and 3 resulted in higher final biomass (9.5 g L^−1^ and 9 g L^−1^) compared to batch 1’s 12-h photoperiod (4.5 g L^−1^).

*G. sulphuraria* cells differ in size at different life cycle stages (Hirooka et al. 2022), affecting cell disruption. Using frozen and thawed biomass, we tested various GB sizes for their ability to produce high amounts of soluble protein (Fig. 1C). Beads of 0.25–0.5 mm produced higher protein yields, 17% with frozen and 30% with thawed, compared to 0.75-to 1-mm beads. Cell lysis was estimated microscopically, with the best results for 0.25- to 0.5-mm beads (90% for frozen, 85% for thawed, Fig. 1D). Further tests with thawed biomass using different GB amounts and a cascade extraction (CAE) of three consecutive cycles showed the best results with a 1:10 (w/w) ratio and 50% protein yield (Fig. 1C, Table 1). Thus, for all subsequent experiments, we used this method to extract native proteins from *G. sulphuraria*. The protein yield of the three *G. sulphuraria* culture batches ranged from 0.37 to 0.39 g g^−1^ DW (Table 1). The quantity of soluble protein in the crude extract (CE) was 2.2 mg mL^−1^ (batch 1), 5.9 mg mL^−1^ (batch 2), and 4.2 mg mL^−1^ (batch 3) with partly huge deviations between extractions (Table 1).

**Table 1:** Amount of soluble protein determined for the respective *Galdieria* extracts after each step of processing. CE, crude extract; HIE, heat-inactivated crude extract; ASE, protein concentrated by ammonium sulfate precipitation; HIASE, heat-inactivated ASE. Total protein: values are given as mean of 16 technical replicates. Soluble protein: values represent the mean of 3–8 technical replicates, depending on the amount of biomass available

		Batch 1	Batch 2	Batch 3
Total protein g g^−1^ DW	Yield	0.38 ± 0.02	0.39 ± 0.03	0.37 ± 0.02
Soluble protein (mg mL^−1^)	CE	2.2 ± 0.5 (*n* = 7)	5.9 ± 0.6 (*n* = 6)	4.2 ± 1.0 (*n* = 6)
	HIE	1.5 ± 0.5 (*n* = 7)	2.6 ± 0.9 (*n* = 3)	3.7 ± 0.6 (*n* = 4)
	ASE	4.9 ± 1.9 (*n* = 5)	6.7 ± 5.3 (*n* = 4)	5.2 ± 0.4 (*n* = 3)
	HIASE	2.4 ± 1.6 (*n* = 8)	5.8 ± 3.8 (*n* = 3)	3.0 ± 0.4 (*n* = 3)

Since FBS is commonly heat-treated (55–60 °C for 10–30 min) to inactivate complement proteins of the innate immune system (ThermoFisher 2025), we did the same for all algae samples. A heat inactivation (HI) resulted in less soluble protein being present in the samples (Table 1). For a concentrated native protein fraction, an ammonium sulfate precipitation was conducted to separate proteins from other cellular components. The precipitated protein fraction (ASE) was resuspended, desalted, and the soluble protein concentration determined. ASE had slightly higher concentrations compared to CE, with 4.9 mg mL^−1^ (batch 1), 6.7 mg mL^−1^ (batch 2), and 5.2 mg mL^−1^ (batch 3). Heat-treated samples (HIASE) had lower protein contents of 2.4 mg mL^−1^ (batch 1), 5.8 mg mL^−1^ (batch 2), and 3 mg mL^−1^ (batch 3).

### Enzyme activity as a measure of native protein

**Fig. 2:**
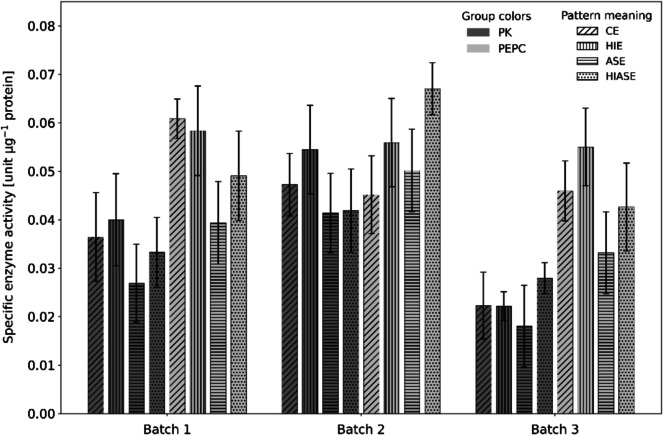
Specific enzyme activity of pyruvate kinase (PK) and phosphoenolpyruvate carboxylase (PEPC). Activity was measured for three distinct batches (batch 1, batch 2, batch 3) of crude extracts (CE), heat-inactivated extracts (HIE), ammonium sulfate extract (ASE), and heat-inactivated ammonium sulfate extracts (HIASE). Bars represent the enzyme activity (units µg.^−1^ protein), with PK groups shown in grey and PEPC groups in light grey. Data present a mean ± standard deviation with *n* = 18

FBS is a complex biological product, and most of the known molecules active in cell culture are native proteins and peptides. Consequently, we decided to extract native protein from *Galdieria* and confirm the nativity of extracts by determining the specific activity of two marker enzymes, PK and PEPC. At least two PK isozymes exist for plants and microalgae: a cytosolic and a chloroplast one, both catalyzing the dephosphorylation of PEP to pyruvate (Unterlander et al. 2017). PEPC resides in the cytosol and is involved in carbon fixation and regulation by catalyzing the carboxylation of pyruvate with bicarbonate (HCO_3_^−^) to oxaloacetate and inorganic phosphorus (e.g., Wang et al. 2017). PK activity was relatively similar across samples within each batch, despite heat treatment, indicating the native nature of the protein samples (Fig. 2). ASE protein extracts generally had lower enzymatic activity. In batch 1, PK activity ranged from 0.036 units µg^−1^ protein in CE to 0.033 units µg^−1^ protein in HIASE. For batch 2, PK activity ranged from 0.047 units µg^−1^ protein in CE to 0.042 units µg^−1^ protein in HIASE. Batch 3 had the lowest PK activity, from 0.02 units µg^−1^ protein in CE to 0.03 units µg^−1^ protein in HIASE. The different crude and protein extracts did not show a loss of PEPC activity within each batch. In all three batches, PEPC activity in CE was above 0.045 units µg^−1^ protein, in HIE above 0.5 units µg^−1^ protein, and in ASE and HIASE around 0.3 and 0.4 units µg^−1^ protein, respectively (Fig. 1D).

### Assessing the effects of algal proteins on CHO cell growth

**Fig. 3:**
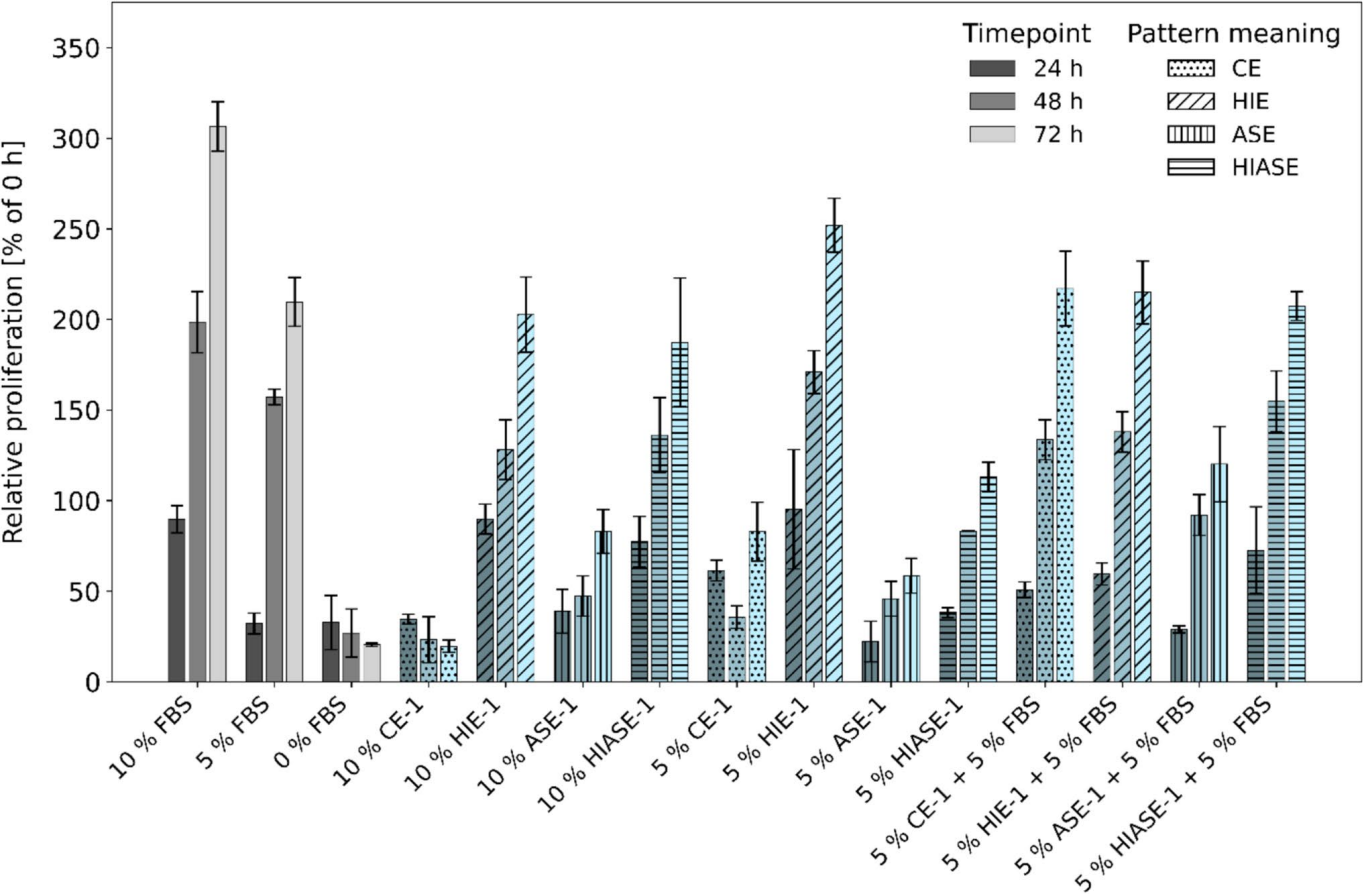
Proliferative effect of native protein extracts from batch 1 of *G. sulphuraria* 074G on CHO cell proliferation. Cells were treated with 10% FBS, 5% FBS, 0% FBS, CE, HIE, ASE, HIASE, and 5% extract + 5% FBS from batch 1. Relative proliferation was calculated as a percentage by setting absorbance values at 24 h, 48 h, and 72 h in relation to the initial value. Results are shown as relative cell proliferation in % ± standard deviation with *n* = 4

The viability of CHO cells was tested using *Galdieria*-derived protein fractions as a FBS replacement over 24, 48, and 72 h. CHO cells were directly inoculated into the serum-free medium without acclimation, and their proliferation rate was monitored via MTT assay and confirmed by cell counts and microscopy (supplemental data). Cells were supplemented with 5% and 10% (v/v) protein samples (CE, HIE, ASE, HIASE) from batches 1–3, and the results were compared to control cultures containing 5% and 10% FBS. CHO cells grew successfully in serum-free medium supplemented with *Galdieria* extracts from culture batch 1 (Fig. 3). Heat inactivation of extracts (HIE-1, HIASE-1) supported viable proliferation comparable to control cultures with 5% and 10% FBS. In contrast, native protein extracts CE-1 and ASE-1 did not induce cell growth. While 5% HIE-1 promoted CHO growth more than 10% HIE-1, the opposite was seen with 5% and 10% HIASE-1. The addition of 5% FBS to 5% CE-1 and 5% ASE-1 resulted in a twofold increase in the CHO proliferation rate. Adding 5% FBS to 5% HIE slightly reduced growth, while adding 5% FBS to 5% HIASE increased cell growth. The amount of protein in the respective samples was 1.5- to twofold higher in CE-1 and ASE-1 compared to HIE-1 and HIASE-1 (Table 2), and the addition of 5% FBS to 5% samples increased the amount of protein in the CHO treatments by about tenfold.

**Table 2:** Amount of soluble protein (g L^−1^) in the respective *Galdieria* extracts (CE-1, HIE-1, ASE-1, and HIASE-1) and soluble protein (µg) in the proliferation medium for CHO cells to determine the viability by MTT assay. For the addition of 10% and 5% of extracts, 10 µL and 5 µL were mixed into a total reaction volume of 100 µL

	**Soluble protein (g L**^**−1**^**)**	**Soluble protein (µg)**		
	Total	10% extract	5% extract	5% extract with 5% FBS
CE-1	2.5	25	13	143
HIE-1	1.7	17	9	139
ASE-1	4.7	49	25	155
HIASE-1	2.2	22	11	141

**Fig. 4:**
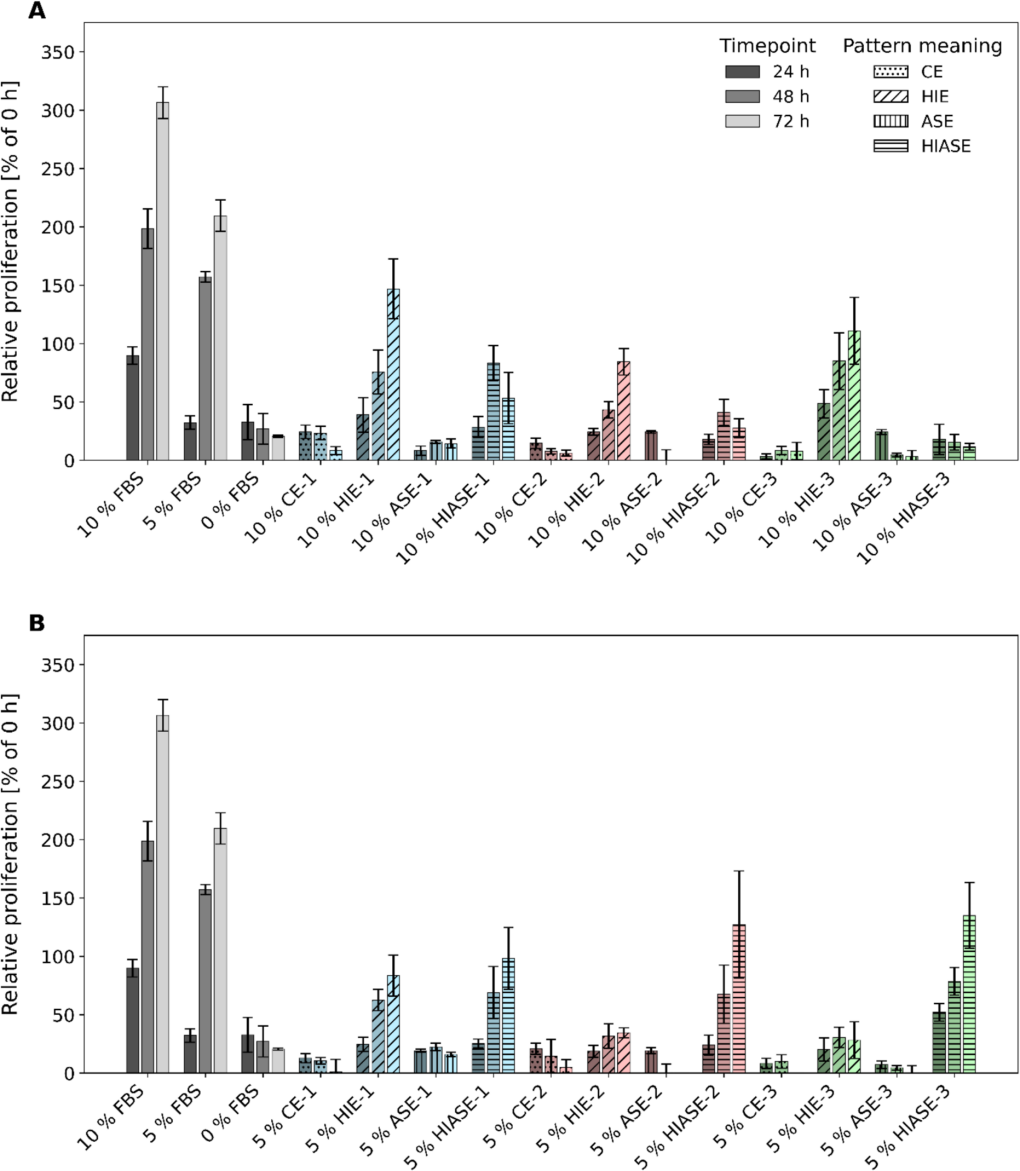
Proliferative effect of native protein extracts from *G. sulphuraria* 074G on cell proliferation of CHO cells. The cells were treated with 10% FBS, 5% FBS, and 0% FBS, as well as 10% (**A**) and 5% (**B**) of the algae extracts in the form of crude extracts (CE), heat-inactivated extracts (HIE) ammonium sulfate extracts (ASE), and heat-inactivated ammonium sulfate extracts (HIASE) from three different batches (1, 2, 3). The relative proliferation was calculated as a percentage by setting the measured absorbance values at the respective time points (24 h, 48 h, and 72 h) in relation to the initial value determined before the start of treatment. Results are shown as relative cell proliferation in % ± standard deviation with *n* = 18

The data were generated from one extraction of biomass from batch 1 and should be replicated in the following. We generated native protein extracts from three *Galdieria* batches, producing four independent extracts from each batch. CHO cells successfully grew in serum-free medium with *Galdieria* extracts, but on average at rates five- to twofold lower than control cultures (Fig. 4A, B). Extracts from the three batches varied significantly, with batch 1 being the most consistent. Heat treatment of protein extracts was confirmed to be necessary for proliferation, e.g., 10% HIE and HIASE of batch 1 significantly increased proliferation, while 10% CE and ASE had no effect (Fig. 4A). The effect of HI was more evident at 5%, where HIASE better promoted growth than HIE (Fig. 4B). The considerable variation in the proliferation rate between samples might be explained by differences in the composition of the protein extracts and the amount of protein. *Galdieria* extracts had 5–10 times lower protein content than FBS, with 248 µg (10% approach) and 130 µg (5% approach, Table 3). The higher proliferation rate with 10% HIE and HIASE (batch 1) compared to 5% suggests protein concentration may account for the reduced rate versus the FBS control.

**Table 3:** Amount of soluble protein (µg) in the proliferation medium for CHO cells to determine the viability by MTT assay. For the addition of 10% and 5% of extracts, 10 µL and 5 µL were mixed into a total reaction volume of 100 µL

	Control		Batch 1		Batch 2		Batch 3	
	Soluble protein (µg) in 10% extract and 5% extract							
FBS	248 ± 7	130 ± 3						
CE			22 ± 5	11 ± 3	59 ± 7	29 ± 4	42 ± 1	21 ± 1
HIE			15 ± 3	8 ± 2	37 ± 9	19 ± 5	37 ± 6	19 ± 3
ASE			49 ± 20	24 ± 10	67 ± 44	33 ± 22	52 ± 5	26 ± 3
HIASE			24 ± 16	12 ± 8	58 ± 38	29 ± 19	30 ± 4	15 ± 2

## Discussion

In this study, we present a production process for obtaining native protein from wet biomass of *G. sulphuraria* 074G, which was intended as an FBS substitute. The protein extracts were tested on serum-free CHO cell cultures and showed proliferative activity after HI. To our best knowledge, this is the first time that several independent extractions of three different cultivation batches were used to evaluate the potential of native protein from *G. sulphuraria* as an FBS replacement.

We first produced algae biomass mixotrophically in a PBR and were able to harvest up to 9.5 g L^−1^ of dry *G. sulphuraria* 074G biomass within 5 days. This was higher than reported for other mixotrophic batch cultivations with the same strain and is possibly explained by the PBR system and general growth conditions (Graverholt and Eriksen 2007; Abiusi et al. 2022). We found that harvesting in the late exponential to linear growth phase yielded higher soluble protein compared to high cell densities. The protein content and extractability are affected by older cultures producing more polymers that trap soluble proteins, causing precipitation after cell disruption and leaving membrane-associated proteins insoluble. Besides culture age, the chemical and physical properties of the cell wall impact the success of cell disruption and protein solubility (Safi et al. 2013). The cell wall of *Galdieria* species is quite rigid, and the closely related *Cyanidium caldarium* has up to 55% proteins in the cell wall (Bailey and Staehelin 1968). *Galdieria* cells are commonly disrupted using a bead mill, high-pressure homogenizer (HPH), or French press (Carfagna et al. 2018; Imbimbo et al. 2019; Abiusi et al. 2022). We found bead milling and HPH to be efficient; however, HPH resulted in more suspended matter in the cell extract (data not shown). Thus, we decided on bead milling. Despite 90% of cells being lysed, protein solubility remained an obstacle. Various extraction matrices were tested, but a fraction of protein remained insoluble. A potential solution could be cascade extraction, which might also allow the extraction of additional cellular fractions (Imbimbo et al. 2019).

The protein data on mixotrophic *Galdieria* cultures show great variability and range from 22 to 72% proteins of DW (Graziani et al. 2013; Wan et al. 2016; Massa et al. 2019; Abiusi et al. 2022; Canelli et al. 2023). In addition to the different *Galdieria* strains that were evaluated, the quantification methods employed are the main reason for large deviations (Barbarino and Lourenço 2005; Abiusi et al. 2022). Bradford and Lowry assays are commonly used, with Lowry providing consistent results and Bradford usually giving lower values (Barbarino and Lourenço 2005), in our case with a factor of 2.39. We used Bradford to quantify the proteins in the extracts, while the total protein was measured using the Lowry assay.

In initial experiments, the proliferation rate of CHO cells in the presence of HI protein extracts matched that of FBS controls. To exclude artifacts from a single experiment, we repeated the whole process with three additional extractions from three independent culture batches. An admixture of 5% of the enriched protein samples (HIASE) appeared to promote CHO growth better than crude extracts (HIE), indicating either a reduction of growth-inhibiting factors or an enrichment of growth-promoting factors. The effect could only be replicated with the 10% batch 1 approach, supporting the hypothesis of a non-uniform protein and peptide composition in the extracts. The protein concentration of *Galdieria* extracts tested was around five- to tenfold lower than that of FBS. However, with 8–37 µg protein in HIE and 12–58 µg protein in HIASE supplemented to serum-free medium, the CHO proliferation rate was significantly higher than 0% FBS cultures, but around twofold lower than the respective 5 and 10% FBS cultures. Either the lower protein levels were the cause of the reduced proliferation rate, or *Galdieria* extracts do not completely replace FBS, and additional growth-promoting molecules should be added to the serum-free medium. If we look at the results of the first test of batch 1, growth of the CHO cells, like FBS controls, seems to be possible even without further additives. Therefore, the identification of the protein and peptide composition of the different extracts seems to be a reasonable solution. In addition, an acclimation of CHO cells to *Galdieria* extracts prior to the actual tests might also improve results. Algae extracts with 1 µg mL^−1^ and 10 µg mL^−1^ protein content showed similar or slightly higher cell viability of myoblast cells from Japanese quail (QM7) when supplemented with 1% FBS (Sibinčić et al. 2024). Higher protein concentrations of algae extracts led to growth-inhibiting effects for most samples. CW of *C. littorale* (15 mg protein mL^−1^) was efficient in replacing FBS in the serum-free culture medium of CHO and other tested cell lines, with similar or slightly lower viabilities than controls (Ghosh et al. 2023). However, this was shown for one extract with three replicates in total.

Supplementing algae extracts with growth factors or low FBS amounts promoted cell growth significantly (Ng et al. 2020; Defendi-Cho and Gould 2023; Dong et al. 2023; 2024; Sibinčić et al. 2024). CVE and CGF showed up to 80% growth-promoting activity in serum-free media with added growth factors such as basic fibroblast growth factor (FGF), transforming growth factors (TGF)-β1, and insulin (Defendi-Cho and Gould 2023). Similar viability and proliferation were observed in fish and mammalian cell cultures using water extracts in serum-reduced medium (Dong et al. 2023; 2024; Sibinčić et al. 2024). Furthermore, phycocyanin C (C-PC), which was present in our extracts, was found to positively regulate the cell cycle of human fibroblast WI-38 cells (Madhyastha et al. 2006; Dranseikienė et al. 2022) and promoted growth in the mouse myoblast cell line C2 C12 and QM7 cells in serum-free medium (Ghosh et al. 2023). All findings indicate the protein fraction of microalgae is key in replacing FBS, making the identification of effective molecules in protein extracts crucial.

The cultured meat and fish industry seeks serum-free media to reduce costs and address ethical and regulatory concerns associated with FBS. Currently, serum-free media account for about 50% of the variable operating costs in cultivated meat production, with growth factors and recombinant proteins being the primary cost drivers (Quek et al. 2024). Microalgae present a promising cost-effective alternative; however, it is crucial to establish an affordable production process for microalgal serum, considering the demand for FBS. Mixotrophic or heterotrophic production with *Galdieria* can be advantageous, as its preference for a very low pH reduces the risk of contamination under heterotrophic conditions compared to, e.g., *Chlorella*, and it contains PC, which is discussed as a key substance (Hirooka and Miyagishima 2016; Dranseikienė et al. 2022). Nevertheless, continuous passage of CHO and other mammalian cell lines on the *Galdieria* protein extracts HIE and HIASE is necessary to prove long-term feasibility. Thus, improving the composition and quality of native protein extracts requires an improved extraction method from high-density *Galdieria* cultures.

## Supplementary Information

Below is the link to the electronic supplementary material.

Supplementary file1 (PDF 1209 KB)

## Data Availability

No datasets were generated or analysed during the current study.
