# Supplementary material for: Native proteins from Galdieria sulphuraria to replace fetal bovine serum in mammalian cell culture
Source: Appl Microbiol Biotechnol. 2025 May 10;109:119. doi: 10.1007/s00253-025-13507-0 (PMC12065734; doi:10.1007/s00253-025-13507-0)
Supplement: Supplementary file 1 [file 253_2025_13507_MOESM1_ESM.pdf]

Native proteins from *Galdieria sulphuraria* to replace fetal bovine serum in mammalian cell culture

Applied Microbiology and Biotechnology

Hanna Eisenberg, Svenja Hütker, Felicitas Berger, Imke Lang

Bremerhaven University of Applied Sciences, Institute EcoMaterials, An der Karlstadt 8, 27568 Bremerhaven, Germany
Corresponding author: ilang@hs-bremerhaven.de

Imke Lang: https://orcid.org/0000-0002-5402-461X
Hanna Eisenberg: https://orcid.org/0009-0005-9575-3949

Supplemental data

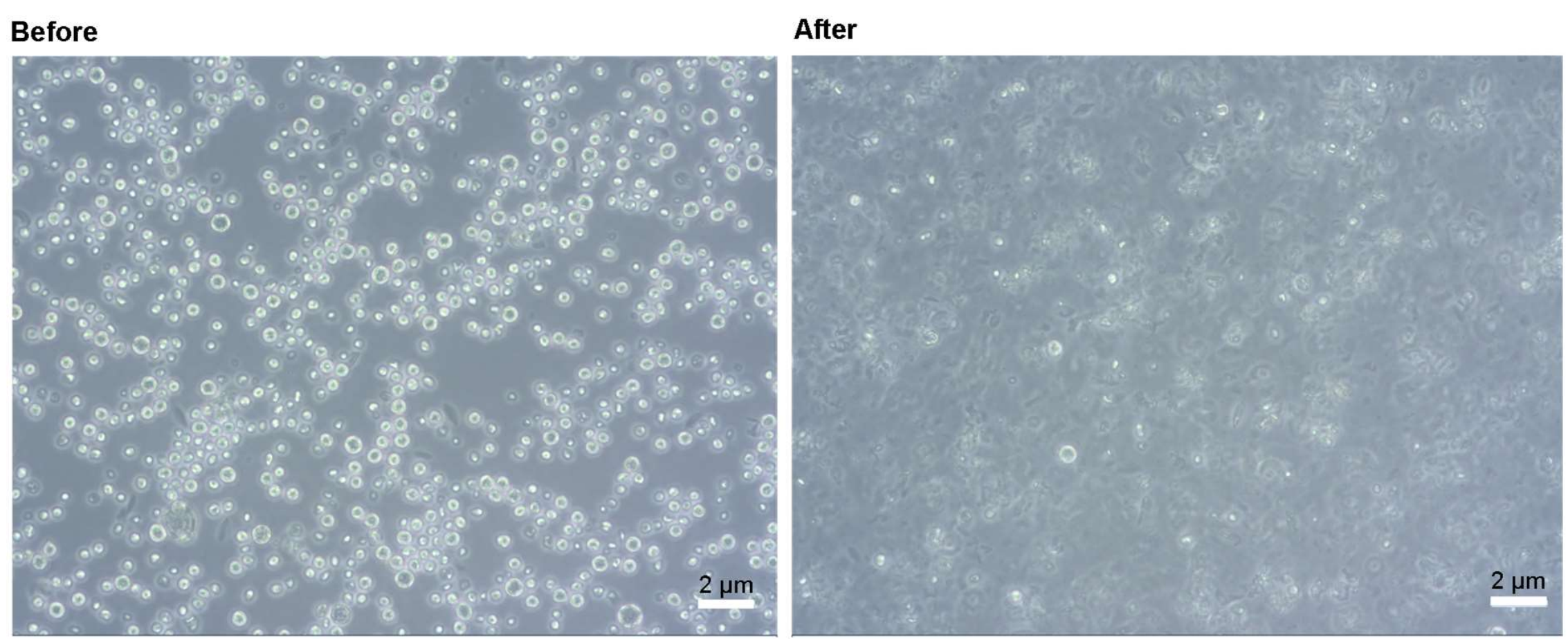

Fig. S1: Microscopic images (400 x magnification, Zeiss Primo Star) of *G. sulphuraria* culture material before (left) and after (right) treatment with six consecutive rounds of bead milling.

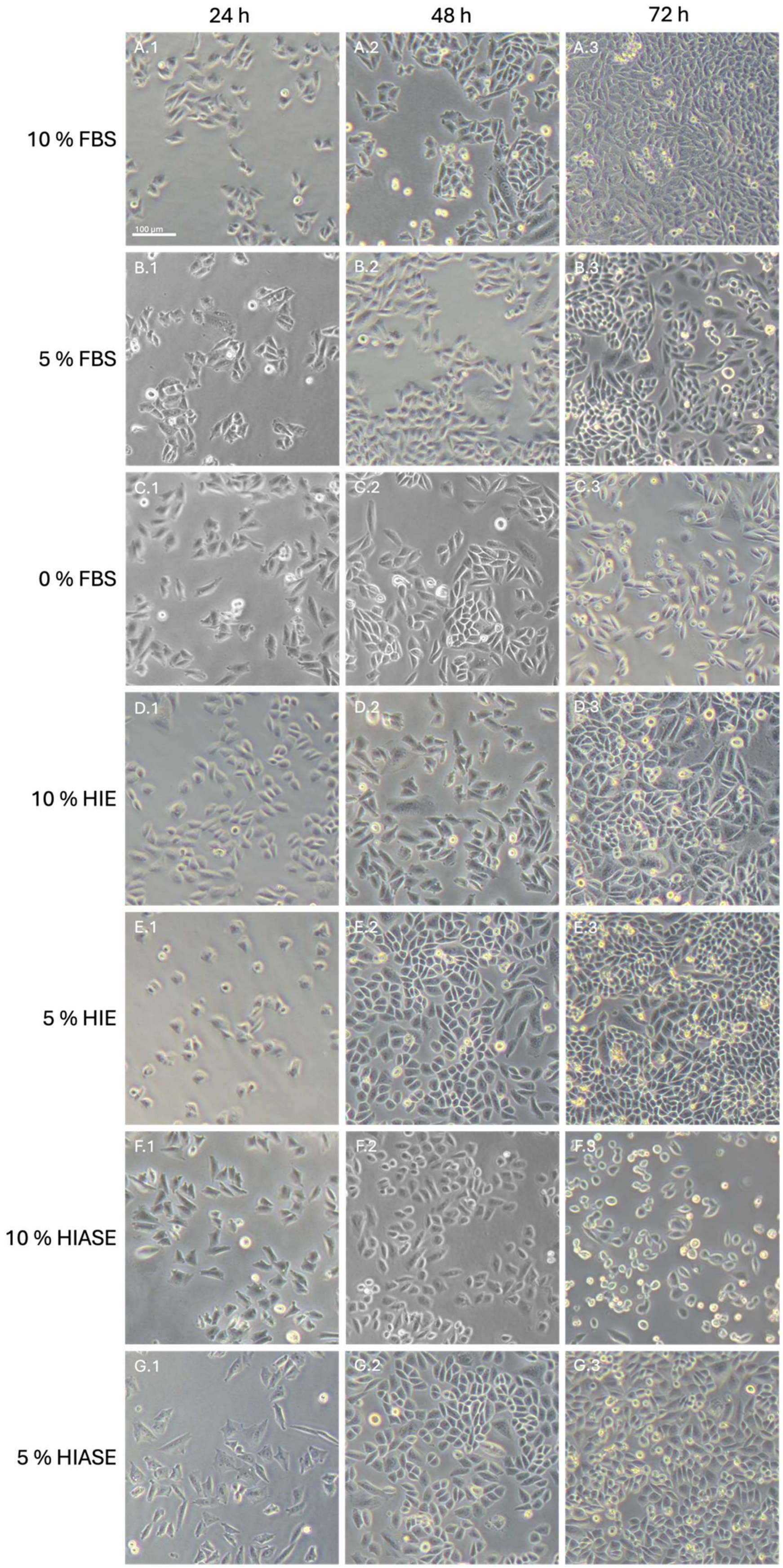

Fig. S2: Microscopic images (1000x magnification, Zeiss Primovert) of CHO cells after treatment with different serum and microalgae extract conditions for 72 hours. Cells were cultured in presence of 10 % FBS (A), 5 % FBS (B), 0 % FBS (C), 10 % HIE (Heat inactivated extract, D), 5 % HIE (E), 10 % HIASE (heat inactivated, F) or 5 % HIASE (G). The images show the cellular morphology after 24 h (1), 48 h (2) and 72 h (3) of cultivation.

Table S1: Cell viability in percent (mean ± standard deviation) at 24h, 48h and 72h after treatment with different serum and concentrations, calculated as the percentage of live cells in the total cell count per condition. For this, cells were mixed in a 1:1 (v/v) ratio with 0.4% trypan blue solution (Sigma Aldrich). To determine cell viability, the stained samples were loaded onto a dual-chamber hemocytometer (Roth) and examined using a light microscope at 100x magnification. The total number of cells, including both unstained (viable) and blue-stained (non-viable) cells, was counted. The groups tested include fetal bovine serum (FBS), heat-inactivated crude extracts (HIE) and heat-inactivated ammonium sulfate extracts (HIASE) at various concentrations. The data were obtained from three biological replicates, each with four technical replicates.

	Viability (%) 24h	Viability (%) 48h	Viability (%) 72h
10% FBS	98.96 ± 0.91	99.22 ± 1.26	99.28 ± 0,25
5% FBS	97.60 ± 0.54	98.87 ± 0,65	99.59 ± 0,97
0% FBS	98.52 ± 1.37	98.46 ± 0,83	98.60 ± 1.78
10% HIE	98.31 ± 0.70	93.65 ± 4.56	96.49 ± 2.78
5% HIE	96.72 ± 0.88	98.20 ± 0.93	96.91 ± 3.98
10% HIASE	9879 ± 0.98	98.93 ± 1.09	87.07 ± 4.37
5% HIASE	98.65 ± 0.91	97.98 ± 1.40	93.53 ± 1.18

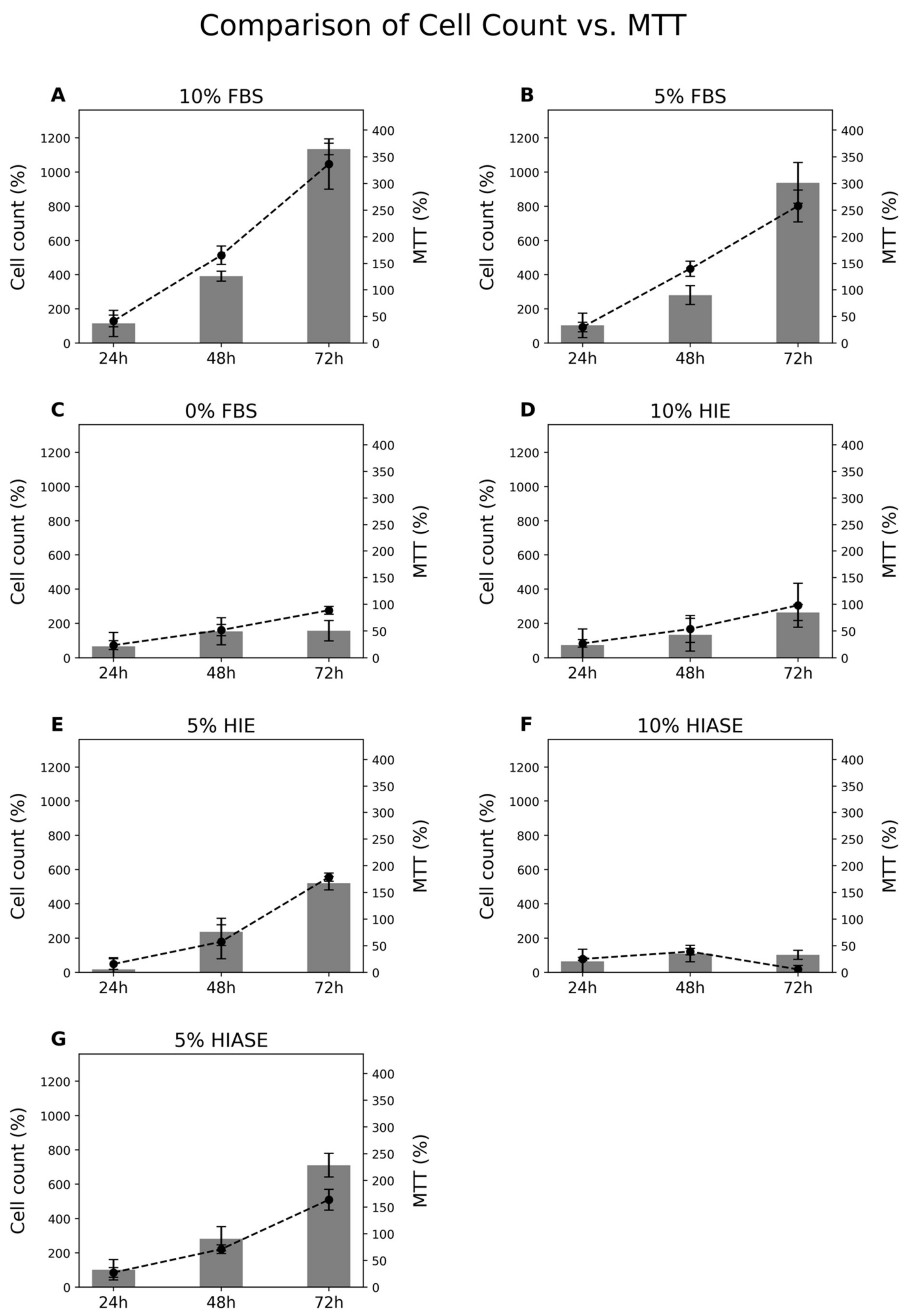

Fig. S3: Comparison of cell proliferation by direct cell counting with a dual-chamber hemocytometer (grey bars, left y-axis) and metabolic activity by MTT assay (dotted line, right y-axis) over the time points 24h, 48h and 72h. Each subplot represents a different cultivation condition: fetal bovine serum (FBS), heat inactivated crude extracts (HIE) and heat inactivated ammonium sulfate extracts (HIASE) at different concentrations. Data are presented as mean ± standard deviation from three biological replicates, each with four technical replicates. Values are expressed as percentage increase compared to the respective baseline value at 0h (100%). The y-axes were scaled to allow comparability of proliferation trends.
